# On the Applicability of Temperature and Precipitation Data from CMIP3 for China

**DOI:** 10.1371/journal.pone.0044659

**Published:** 2012-09-19

**Authors:** Chiyuan Miao, Qingyun Duan, Lin Yang, Alistair G. L. Borthwick

**Affiliations:** 1 State Key Laboratory of Earth Surface Processes and Resource Ecology, College of Global Change and Earth System Science, Beijing Normal University, Beijing, People's Republic of China; 2 State Key Laboratory of Resources and Environmental Information System, Institute of Geographical Sciences and Natural Resources Research, Chinese Academy of Sciences, Beijing, People's Republic of China; 3 Department of Civil & Environmental Engineering, University College Cork, Cork, Ireland; University of Oxford, United Kingdom

## Abstract

Global Circulation Models (GCMs) contributed to the Intergovernmental Panel on Climate Change (IPCC) Fourth Assessment Report (AR4) and are widely used in global change research. This paper assesses the performance of the AR4 GCMs in simulating precipitation and temperature in China from 1960 to 1999 by comparison with observed data, using system bias (*B*), root-mean-square error (*RMSE*), Pearson correlation coefficient (*R*) and Nash-Sutcliffe model efficiency (*E*) metrics. Probability density functions (PDFs) are also fitted to the outputs of each model. It is shown that the performance of each GCM varies to different degrees across China. Based on the skill score derived from the four metrics, it is suggested that GCM 15 (ipsl_cm4) and GCM 3 (cccma_cgcm_t63) provide the best representations of temperature and precipitation, respectively, in terms of spatial distribution and trend over 10 years. The results also indicate that users should apply carefully the results of annual precipitation and annual temperature generated by AR4 GCMs in China due to poor performance. At a finer scale, the four metrics are also used to obtain best fit scores for ten river basins covering mainland China. Further research is proposed to improve the simulation accuracy of the AR4 GCMs regarding China.

## Introduction

The global temperature has significantly increased in recent decades, according to both direct measurements and credible proxy data. The IPCC-AR4 indicates that the rise in global-average surface temperature has been particularly pronounced since about 1950, with an updated trend of 0.74±0.18 °C during 1906–2005 [1]. IPCC-AR4 also estimates that on average a warming of about 0.2 °C/decade may occur during the next two decades. Another key variable, precipitation is expected to increase under global warming at high latitudes and in the vicinity of the equator, but decrease in the subtropics [2]. It is believed that precipitation will experience an overall increase on average due to there being greater evaporation [3]. Climate change can have major impacts on vulnerable natural systems and sensitive human systems at local, regional and national scales. Accordingly, there is an urgent need for an improved understanding of climate change, its consequences, and mitigation and adaptation strategies [4].

To provide information to support IPCC-AR4, more than 20 modeling groups around the world conducted climate change simulations using different GCMs. These IPCC-AR4 GCMs can be used to simulate present-day and projected future climate conditions under different scenarios, and hence inform decision makers regarding potential mitigation measures and adaptation strategies. However, the theoretical description of the climate remains incomplete, and simplifying assumptions are inherent when building these GCMs [5]. Epistemic and aleatory uncertainties in climate models introduce biases into the simulations, and so GCMs are unable to represent fully the intensity and frequency of observed data on climate characteristics [6]–[8]. Several researchers have assessed the performance of GCMs from the global [5], national [9], [10] and regional [11], [12] scales respectively. The results have demonstrated that not all GCMs are able to provide a similarly accurate description of the present climate [13]–[18]. Furthermore, it should be noted that the performance of the AR4 GCMs is not uniformly consistent over large geographical areas, especially for the extreme climate variables [11], [12]. Consequently, the accuracy of any GCM should be established through validation studies before using it to predict future climate scenarios [19]. Although accurate simulation of the present climate does not guarantee that forecasts of future climate will be reliable [5], it is generally accepted that the agreement of model predictions with present observations is a necessary prerequisite in order to have confidence in the quality of a model [17], and models that reproduce accurately the present climate are more likely to provide reasonably accurate predictions of future climate [20].

China has experienced gradual warming throughout the 20th Century consistent with the warming observed at global scale. It was reported that the mean annual surface air temperature in mainland China increased by about 1.3 °C from 1951 to 2004 due to the greenhouse effect and rapid urbanization [21]; the warming rate of about 0.25 °C/decade is more than twice the global warming rate. No significant trend in mean precipitation occurred during this period taking China as a whole [22], however, the North and northeast regions experienced a 12% decline in precipitation from 1960 to 2005 while the South had increasing rainfall during the summer and winter seasons [23] due to the East Asian Monsoon variability [24]–[26]. In short, the climate in China varies considerably in space and time due to the scale and complexity of its land topography [27].

The present paper aims to assess the performance of the IPCC-AR4 GCMs in the simulation of precipitation and temperature throughout mainland China (excluding Taiwan island) from the spatial scale (country and large river basin) and temporal scale (intra- and inter- annual) respectively.

## Materials and Methods

### Data

**Figure 1:**
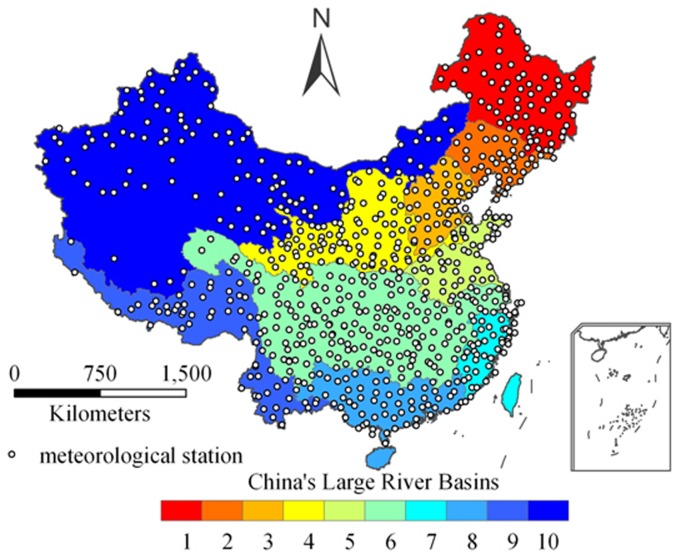
Locations of meteorological stations and major river basins in mainland China. The color coding relates to the following river basins: 1 Songhua River; 2 Liaohe River; 3 Haihe River; 4 Yellow River; 5 Huaihe River; 6 Yangtze River; 7 southeast drainage area rivers; 8 Pearl River; 9 southwest drainage area rivers; 10 northwest drainage area rivers.

Observed data on surface air temperature and precipitation for the period from 1960 to 1999 were obtained from the National Meteorological Information Center, China Meteorological Administration. Daily measurements of daily temperature and precipitation were acquired from a total of 731 meteorological stations (Figure 1), and subjected to quality control processes including homogenization, cross-validation, and topographic correction [28]. Following Chinese Bureau of Meteorology Standards, monthly and annual climatic datasets were derived from daily data, and interpolated onto a grid at 1°×1°resolution comprising a total of 1023 cells covering mainland China. Further details of the quality control processes and the archived raw data are given by the National Meteorological Information Center (NMIC, available at http://cdc.cma.gov.cn). It should also be noted that the observed data from each meteorological station were interpolated onto the grid with topographic correction provided by a high resolution digital elevation model (DEM). However, topographic corrections were not applied during the grid interpolations of the outputs of the AR4 GCMs, which unavoidably impacts on model accuracy.

Monthly temperature and precipitation simulation data were produced by the 24 AR4 GCMs as part of the Coupled Model Intercomparison Project Phase 3 (CMIP-3) of the World Climate Research Programme (WCRP). The data are stored in a multi-model dataset [29], [30] archived by the Program for Climate Model Diagnosis and Intercomparison (PCMDI, available at https://esg.llnl.gov:8443/index.jsp ). The CMIP-3 data outputs used herein are taken from the Twentieth Century (20C3M) experiment, which has the most realistic forcings. Table 1 lists the various models. Further details of model status, model documentation, related references, etc., are available from the website http://www-pcmdi.llnl.gov/ipcc/info_for_analysts.php. The spatial resolution of the models used in the present analysis varied from 1.125°×1.125° for GCM 16 (miroc3_2_hires model) to 5°×4° for GCM 10 (giss_model_e_h model) [1]. For comparison purpose, all model results were interpolated to the same resolution as that of the observed data (1°×1°grid). All model outputs are taken from a single result (run 1).

**Table 1:** List of the global climate models used in this research.

GCM	Model	Source
1	bccr_bcm2_0	Bjerknes Centre for Climate Research, Norway
2	cccma_cgcm3_1	Canadian Centre for Climate Modelling and Analysis
3	cccma_cgcm_t63	Canadian Centre for Climate Modelling and Analysis
4	cnrm_cm3	Centre National de Recherches Meteorologiques, France
5	csiro_mk3_0	Australian Commonwealth Scientific and Research Org.
6	csiro_mk3_5	Australian Commonwealth Scientific and Research Org.
7	gfdl_cm2_0	Geophysical Fluid Dynamics Laboratory, United States
8	gfdl_cm2_1	Geophysical Fluid Dynamics Laboratory, United States
9	giss_aom	Goddard Institute of Space Studies(NASA), United States
10	giss_model_e_h	Goddard Institute of Space Studies(NASA), United States
11	giss_model_e_r	Goddard Institute of Space Studies(NASA), United States
12	iap_fgoals1_0_g	Institute of Atmospheric Physics, China
13	ingv_echam4	National Institute of Geophysics and Volcanology, Italy
14	inmcm3_0	Institute for Numerical Mathematics, Russia
15	ipsl_cm4	Institut Pierre Simon Laplace, France
16	miroc3_2_hires	Center for Climate System Research, Japan
17	miroc3_2_medres	Center for Climate System Research, Japan
18	miub_echo_g	Meteorological Institute of the University of Bonn, Germany
19	mpi_echam5	Max-Planck-Institute for Meteorology, Germany
20	mri_cgcm2_3_2a	Meteorological Research Institute, Japan
21	ncar_ccsm3_0	NCAR Community Climate System Model, USA
22	ncar_pcm1	NCAR Parallel Climate Model, USA
23	ukmo_hadcm3	Hadley Centre for Climate Prediction, UK
24	ukmo_hadgem1	Hadley Centre for Climate Prediction, UK

### Skill score metrics

In analyzing the simulated temperature and precipitation patterns from 1960 to 1999, four metrics were used to indicate the overall agreement between the predictions *P* from each AR4 GCM and the measured observations *O*. The first metric is the system bias (*B*) between the 40-year mean values of the simulated and observed data:
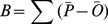
(1)in which the overbar indicates a time-average.

The second metric is the spatially (and temporally) aggregated root-mean-square error (*RMSE*) between the simulated and observed data [7]:
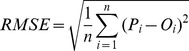
(2)where the summation is taken over a total of *n* spatial grid points (or temporal units). Thus, the smaller is the value of *RMSE*, the closer are the point-wise magnitudes of the simulated and observed climate characteristics.

The third metric is the Pearson correlation coefficient that quantifies similarities between the spatial (and temporal) patterns of the predicted and the observed values:
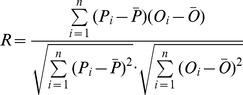
(3)where, again, all summations are over a total of *n* spatial grid points (or temporal units). The Pearson correlation coefficient 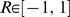, with *R*∼1 implying a close match between the (spatial or temporal) patterns of simulated and observed climate characteristics, and *R*∼0 indicating a lack of similarity. When *R*∼−1, the respective simulated and observed fields are similar in pattern, but their point-wise (spatial or temporal) variations are oppositely signed.

The fourth metric is the Nash-Sutcliffe model efficiency (*E*) [31] that assesses quantitatively the accuracy of the (spatial or temporal) patterns of the model outputs from:
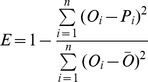
(4)where 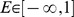. In interpreting the results, it should be noted that *E*∼1 indicates better accuracy, *E* = 0 indicates the predictions have accuracy equal to the mean of the observations, and *E*<0 indicates that the observed mean is better than the model as a predictor [32].

## Results

### System bias analysis

**Figure 2:**
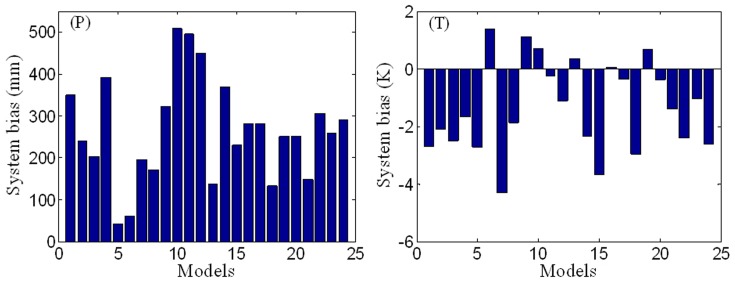
Bar chart indicating the system bias of different AR4 GCMs with regard to annual mean precipitation (*P*) and temperature (*T*) in mainland China during 1960–1999.

Figure 2 shows the system bias for each of the 24 AR4 GCMs obtained by comparing the 40-year overall mean values of the observed and simulated data on annual mean precipitation and temperature. The GCMs give reasonably accurate predictions of the temperature, but are less successful at reproducing the precipitation. All GCMs overestimate precipitation throughout China, with GCM 10 (giss_model_e_h) giving the maximum system bias, which is almost double the annual mean precipitation. It should be noted that the 40-year mean of observed precipitation is 563.1 mm/yr. For temperature, 18 models underestimate the annual mean temperature, and the rest overestimate. The maximum system bias (for temperature simulation) comes from GCM 7 (gfdl_cm2_0) with a value of −4.30 K/yr. GCM 16 (miroc3_2_hires) performs best with a low system bias of 0.065 K/yr.

### Spatial simulations after removal of system bias

**Figure 3:**
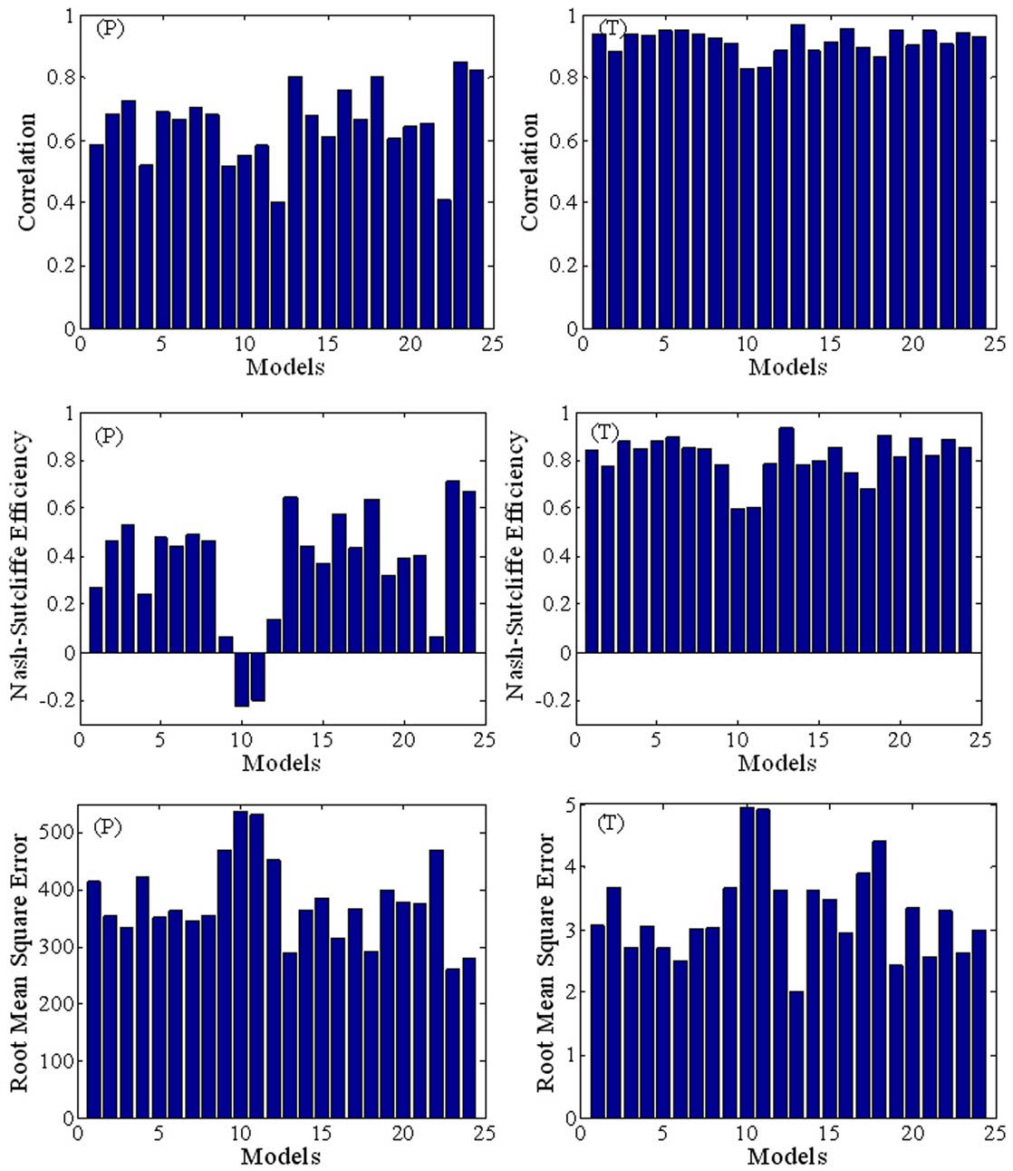
Bar chart indicating relative performances of the AR4 GCMs with regard to the spatial simulation of annual mean precipitation (*P*) mm and temperature (*T*) K in mainland China during 1960–1999.

In order to assess systematically the performance of the AR4 GCMs, the system bias is removed from each dataset by multiplying the monthly model data by the ratio of overall mean model data to overall mean observed data. Figure 3 shows that the calculated spatial correlation coefficients obtained are invariably over 0.5, except for GCM 12 (iap_fgoals1_0_g) and GCM 22 (ncar_pcm1). This indicates that the majority of the AR4 GCMs provide satisfactory simulations of the spatial distribution of annual mean precipitation during 1960–1999. GCM 10 (giss_model_e_h) and GCM 11 (giss_model_e_r) give the poorest simulations comparatively speaking, with *E*<0 and the highest values of *RMSE*. GCM 23 (ukmo_hadcm3) is the best overall at simulating the spatial distribution of annual precipitation (with *R* = 0.85 and *E* = 0.71). Turning to annual mean temperature, the spatial correlation coefficients obtained for the AR4 GCMs are invariably over 0.8, and, in general, it can be seen from Figure 3 that the AR4 GCMs are better at simulating the spatial distribution of annual mean temperature than that of precipitation. Similar to the comparative performance for annual mean precipitation, GCM 10 (giss_model_e_h) and GCM 11 (giss_model_e_r) perform worst spatially for annual mean temperature in terms of *E* and *RMSE*. GCM 13 (ingv_echam4) gives the most accurate results in terms of the spatial distribution of the annual mean temperature data, with *R* = 0.96 and *E* = 0.93.

**Figure 4:**
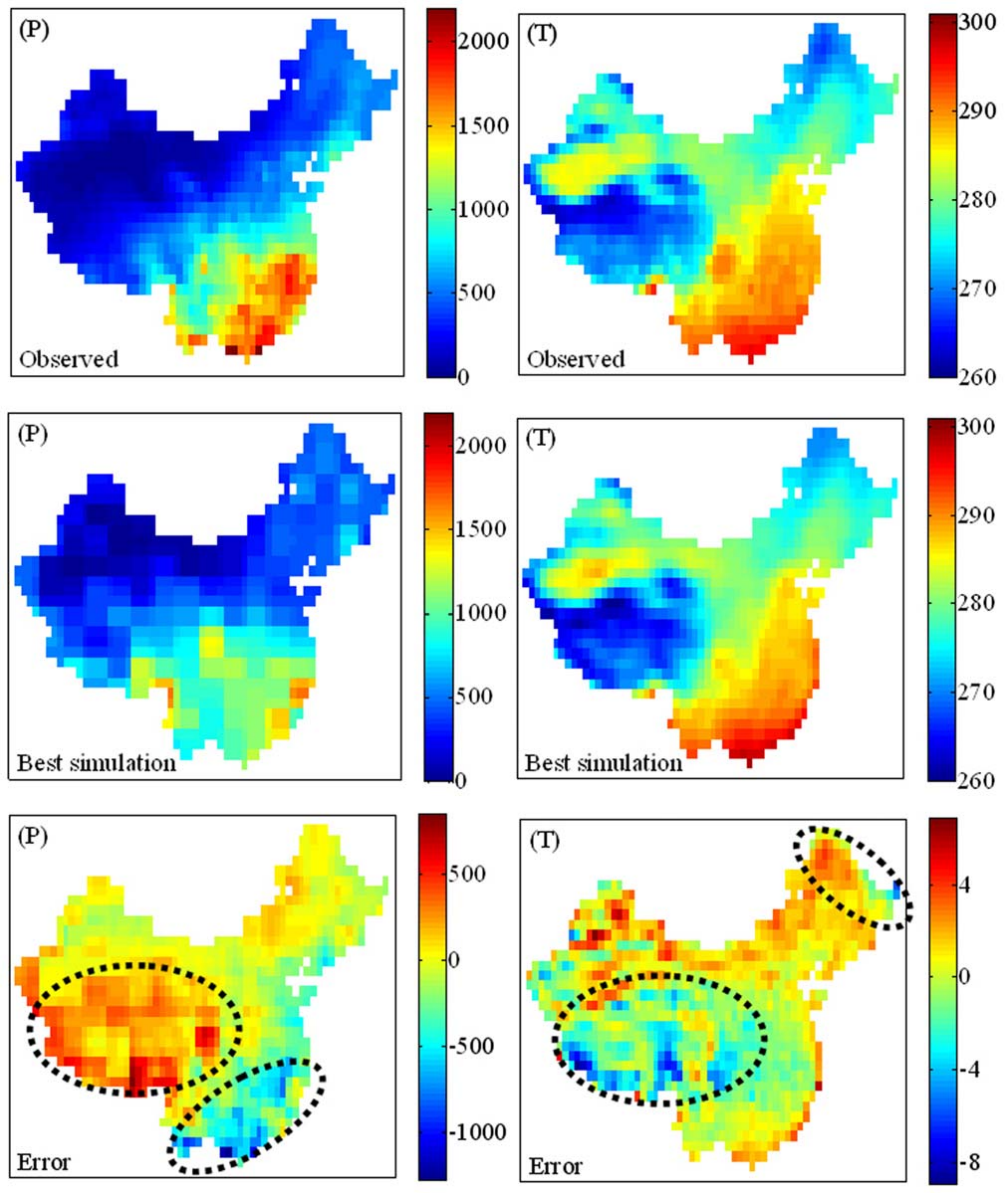
Spatial simulation results for annual mean precipitation (*P*) and temperature (*T*) distributions in mainland China. Best simulation means the best model's simulated results; ukmo_hadcm3 is the best performance precipitation simulation and ingv_echam4 is the best performance temperature simulation; Error is the error between the observed and the best model simulation.

Figure 4 presents the best performance spatial distribution simulations (from GCM 23 - ukmo_hadcm3 for precipitation and GCM 13 - ingv_echam4 for temperature ). The plots confirm that the AR4 GCMs reproduce the important spatial characteristics of precipitation and temperature in mainland China. The climate warm and wet in South China, but cool and dry in northwest China and northeast China. Figure 4 also provides error contours obtained using the best performing models. Regions where the precipitation error is largest are indicated by the superimposed ellipses in southeast China and West China. The maximum error in temperature simulation occurs in northeast China and West China.

### Temporal simulations after removal of system bias

**Figure 5:**
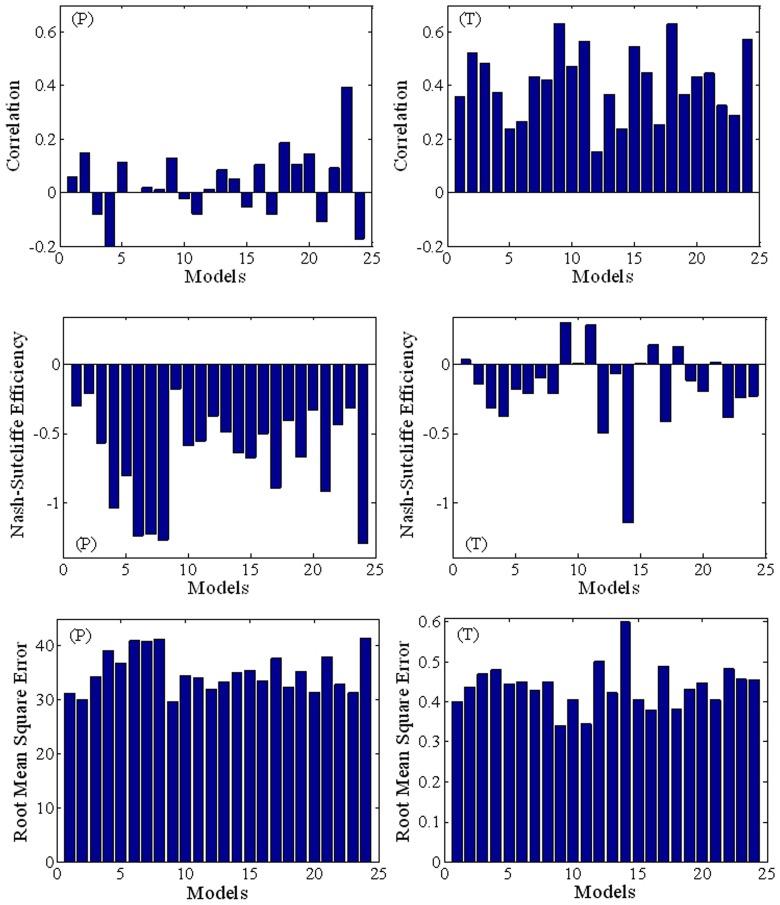
Bar chart indicating relative performances of the AR4 GCMs regarding the temporal simulation of inter- annual mean precipitation (*P*) and temperature (*T*) in mainland China during 1960–1999.

Figure 5 shows the temporal performance of AR4 GCMs in simulating annual mean precipitation and annual mean temperature for mainland China. By comparison with Figure 3, the results presented in Figure 5 show that the GCMs provide less accurate inter-annual temporal than spatial simulations. This is especially the case for precipitation where, for all GCMs, *R*<0.4 and *E*<0, implying that the observed mean is a better predictor than the model. For temperature simulation, *R* is mainly between 0.3–0.5, and *E* remains unacceptable. It is generally accepted that the warming in the late 20^th^ century in AR4 GCMs was likely mainly due to increases of greenhouse gases [9], [33]. Hence, the time series after linear detrending will represent the performance of GCMs in simulating inter-annual variabilities more objective. It is found that the correlation after linear detrending between the observation and the GCM simulation is weaker further (*R*<0.2, *E*<0 for precipitation, and *R*<0.3, *E*<0 for temperature in all GCMs) when comparing with the original time series. Consequently, direct use of the GCM outputs to model the inter-annual variation is not recommended in mainland China, especially for the precipitation simulation.

**Figure 6:**
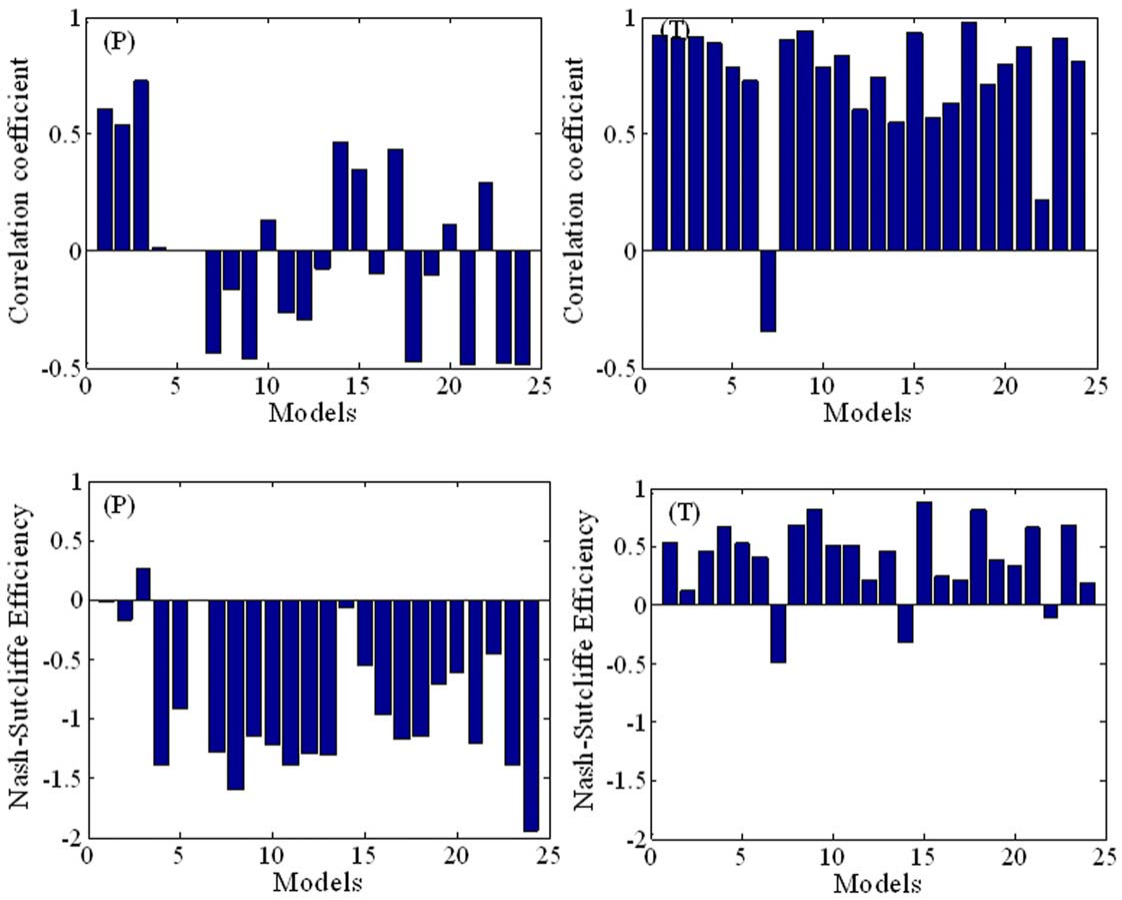
Bar chart indicating relative performances of the AR4 GCMs regarding the temporal simulation of the 10-year moving averages of precipitation (*P*) and temperature (*T*) in mainland China during 1960–1999.

Figure 6 illustrates the performance of the AR4 GCMs in temporal simulation of the 10-year moving average precipitation and temperature. Although the AR4 GCMs are much less accurate at representing the 10-year moving average precipitation than the temperature, the precipitation results are nevertheless considerably improved in comparison with the temporal inter-annual variability of mean precipitation (in Figure 5); and the results from GCM 3 (cccma_cgcm_t63 ) appear to be relatively acceptable. Turning to 10-year moving average temperature, it can be seen that the trend is accurately simulated by all the models except GCM 7 (gfdl_cm2_0) and GCM 22 (ncar_pcm1). GCM 16 (miroc3_2_hires) performs best with *E* = 0.83 and *R* = 0.96. In short, the results imply that the AR4 GCM simulations give an approximate view of the inter-decadal and long-term trends of temperature over China.

**Figure 7:**
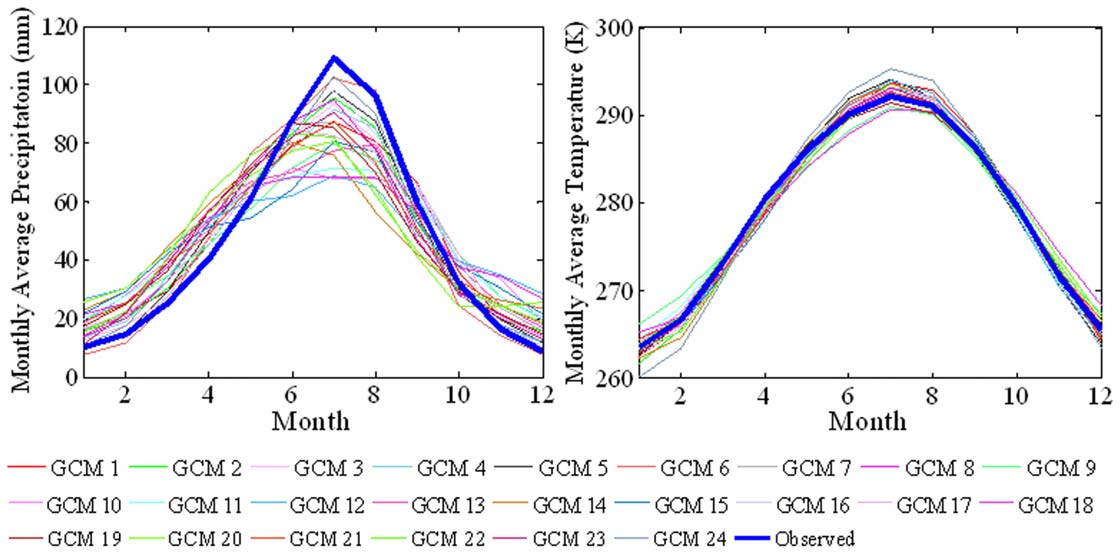
Observed and simulated forty-year averages of monthly precipitation and temperature throughout a calendar year in mainland China during 1960–1999.

Figure 7 plots the simulated and observed forty-year average monthly precipitation and temperature in mainland China. The observed precipitation and temperature results show the steep onset of summer rainfall associated with the summer monsoon, which peaks sharply in July. Almost all the AR4 GCMs succeed in capturing the seasonal variation characteristics of a single peak, except the monthly precipitation simulations by GCMs 10 (giss_model_e_h), 11 (giss_model_e_r) and 18 (miub_echo_g). Almost all the AR4 GCMs overestimate the precipitation in winter and spring, and underestimate the precipitation in summer. The resulting gross estimation error implies that these models are unlikely to be directly useful for hydrological impact assessment. In general, the AR4 GCMs simulate the forty-year average monthly temperature more accurately than the corresponding precipitation. All models capture the bell-shape of the forty-year average monthly temperature profile. However, certain models (i.e. GCM 5 - csiro_mk3_0, GCM 14 - inmcm3_0 and GCM 24 - ukmo_hadgem1) predict temperatures that are too hot in summer and too cold in winter. Other models (such as GCM 19 - giss_aom and GCM 18 miub_echo_g) predict a climate that is too cool in summer and too warm in winter. Overall, GCM 3 (cccma_cgcm_t63) gives the most accurate forty-year average climate simulation.

### Probability density functions after removal of system bias

**Figure 8:**
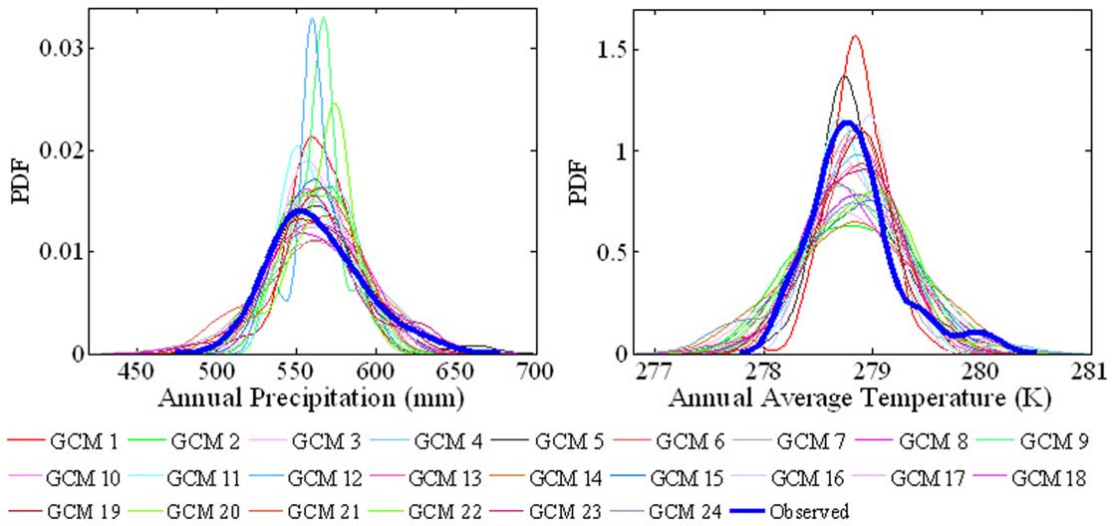
Probability density functions for annual mean precipitation and annual mean temperature in mainland China during 1960–1999.

Figure 8 shows the probability density functions (PDFs) of annual mean precipitation and annual mean temperature. The PDF for the observed annual mean precipitation covers a range from 475 mm to 678 mm, and is slightly skewed (due to the monsoon effect). In general, the simulated PDF for annual mean precipitation is similar but narrower and taller than the observed PDF, especially for GCM 9 (giss_aom) and GCM 20 (mri_cgcm2_3_2a) which give the two highest peaks in the left hand plot of Figure 8. The PDF for observed annual mean temperature ranges from 277.8 K to 280.5 K, and is again asymmetric with a steep rising limb and a broader undular tail. Typically, the AR4 GCMs simulate a wider, lower PDF profile for annual mean temperature that is symmetric and possibly Gaussian, except GCM 1 (bccr_bcm2_0) and GCM 5 (csiro_mk3_0), which give the two largest peaks in the right hand plot of Figure 8.

## Discussion

The present study has demonstrated that the AR4 GCMs exhibit a wide range of performance skills in reproducing the recent (1960–1999) observed climate throughout mainland China. Measured and simulated surface air temperatures and precipitations have been interpreted in terms of spatial distributions, inter-annual and intra-annual trends, and PDFs in order to evaluate the performance of the AR4 GCMs. The results demonstrate that certain models are unsuitable for application to China, with little capacity to simulate the spatial variations in climate across the country. It should be emphasized however that the present conclusions should not be generalized to other climate variables or to other regions of the world.

In general, the simulations are more accurate in space than time, and temperature is better simulated than precipitation. When carrying out research into continental precipitation, it is found that the AR4 GCMs exhibit systematic model bias with most models displaying aggregated precipitation variability magnitudes that are larger than observed [19]. The present study shows similar overestimation to be the case for AR4 GCM simulations of temperature and precipitation in China. The ubiquitous system bias means that caution should be applied when using outputs from the AR4 GCMs in hydrological and ecological assessments..

Several potentially disturbing factors complicate the agreement between the models and reality. The first is model resolution. It is believe that higher resolution does not automatically lead to improved model accuracy [6]. The present research confirms this view. Whereas GCM 13 (ingv_echam4) gives the best spatial temperature simulation, its resolution is only moderate. However, the degree of resolution must have an effect on the accuracy of the spatial simulation of a given model, even after the model outputs have been interpolated onto a grid of uniform resolution (1°×1° in the present study).

The second factor relates to the quality of the observed data. Meteorological stations are non-uniformly distributed in China, with stations particularly sparse in the West and northwest of China. Although interpolation is used to deal with the scarcity of meteorological data in these regions, the results do not properly represent the actual climate due to limitations of the interpolation techniques used. The lack of meteorological stations in the West of China is therefore mainly responsible for the occurrence of the largest errors in this region (Figure 4). On the other hand, the observed climatological records often contain inhomogeneities, which is defined as a change point (a time point in a series such that the observations have a different distribution before and after this time) in the data series [34]. The causes of inhomogeneity can be induced by several non-climatic factors: changes in measurement practices, station relocations, changes in the surroundings of a station over the years, etc. [35]. The homogeneity of observed data has not been detected in this research. If the inhomogeneity is identified and then homogenization techniques are performed to compensate for the biases produced by the inhomogeneities, it is possible potentially improve the agreement of AR4 GCMs with the observations.

The third factor is scale. The AR4 GCMs were used to simulate changes in the climate as a result of slow alterations to certain parameters (such as the greenhouse gas concentration and the solar constant), which affect the energy balance at the global scale. Previous research has shown that data from the AR4 GCMs can accurately reproduce the spatial variations in climate characteristics in Iberia [14], Australia [36], North America [37], and global continents [19]. However, China is a region of particularly complicated topography, with the Tibetan Plateau to the West and various mountain chains in the northern and central regions [27]. China lies mainly in the northern temperate zone and experiences an annual monsoon season. Consequently, China's climate differs considerably from region to region, making accurate local simulation by the AR4 GCMs less likely. This scale discrepancy certainly influences AR4 GCM performance when applied to regions within China.

The fourth factor relates to forcing agents of GCMs. Three possible forcing agents have been identified to contributors to the 20^th^ century global warming [8], [38], mainly includes anthropogenic greenhouse gases forcing (CO_2_, CH_4_, N_2_O, etc.), natural forcing (sulfate aerosols and ozone change) and the internal variability of climate system itself (North Atlantic Oscillation, NAO and E1 Niño – Southern Oscillation, ENSO). Although preexisting researches have suggested the late 20^th^ warming was likely mainly due to increases of greenhouse gases [33], it was reported that the inclusion of natural forcing has improved the simulation [9]. Some GCMs have not included the time-varying natural forcings, such as bccr_bcm_2_0, csiro_mk3_0 etc.. On the other hand, the internal variability of the climatic system is still not full considered in AR4 GCMs. All of these certainly influence the performance of inter-annual simulation. Consequently, AR4 GCMs give unsatisfactory simulations of the inter-annual temporal variability but acceptable inter-decadal variability simulations.

In practice, any user of AR4 GCM data would certainly hope to choose the best model for a particular region, and skill score metrics provide a way of ranking the AR4 GCMs. However, the present study shows that no one model is best at spatial, inter-annual, and intra-annual simulations of both precipitation and temperature. From the results, it is obvious that the inter-annual simulations (temperature and precipitation) by AR4 GCMs are not suitable for direct application. It is recommended that techniques for improving annual simulation should be first investigated. Previous research has indicated that multi-model ensemble simulation can produce better agreement with observed data than any single model [7], [19]. Various ensemble prediction methods have been proposed, including simple model averaging [19], [39], reliability ensemble averaging [40], and Bayesian model averaging [7]. The present study has focused on assessing the performance of each AR4 GCM in assessing certain climate characteristics in China. Multi-model ensemble prediction is recommended as the next step.

Based on comprehensive performance of the models simulation for spatial distribution and inter-decadal trend, we recommend that the best climate models to China are GCM 15 (ipsl_cm4) for temperature, and GCM 3 (cccma_cgcm_t63) for precipitation. As shown in Figure 1, China can be divided into ten large or aggregate river basins comprising the Songhua River, Liaohe River, Haihe River, Yellow River, Huaihe River, Yangtze River, southeast drainage area rivers, Pearl River, southwest drainage area rivers and northwest drainage area rivers. Table 2 lists the models which give the best simulations after 10-year moving average in precipitation and temperature for each basin. The results once again demonstrate that no AR4 GCM performs consistently the best throughout mainland China.

**Table 2:** Top ranked climate model for different river basins.

Precipitation simulation										
Basin	1	2	3	4	5	6	7	8	9	10
GCM	14	12	10	12	19	18	23	22	13	2

Temperature simulation										
Basin	1	2	3	4	5	6	7	8	9	10
GCM	18	24	18	18	18	11	11	15	10	9

The AR4 GCMs do not perform uniformly well in simulating the characteristic spatial and temporal behaviors of temperature and precipitation in China. No one model is best at all simulations. In general, the AR4 GCMs tend to overestimate the precipitation and temperature over China. Furthermore, the model simulations are better at fitting the spatial than the annual temporal behavior, and provide more accurate simulations of temperature than precipitation. By ranking the models according to the four skill score metrics, it has been found that the most appropriate climate models for application to mainland China are GCM 15 (ipsl_cm4) for temperature and GCM 3 (cccma_cgcm_t63) for precipitation. We recommend that AR4 GCM outputs of annual changes in temperature and precipitation are not applied directly to scenarios specific to mainland China. Instead, it is necessary that the data accuracy be improved.

Precipitation and temperature are climate parameters that directly affect hydrological processes, agricultural production, ecosystem restoration, and environmental protection in China. At river basin scale, GCM 14 (inmcm3_0), GCM 12 (iap_fgoals1_0_g), GCM 10 (giss_model_e_h), GCM 12 (iap_fgoals1_0_g), GCM 19 (mpi_echam5), GCM 18 (miub_echo_g), GCM 23 (ukmo_hadcm3), GCM 22 (ncar_pcm1), GCM 13 (ingv_echam4) and GCM 2 (cccma_cgcm3_1) provide the best results in simulating the inter-decadal precipitation trends in the Songhua River, Liaohe River, Haihe River, Yellow River, Huaihe River, Yangtze River, southeast drainage area rivers, Pearl River, southwest drainage area rivers and northwest drainage area rivers respectively. GCM 18 (miub_echo_g), GCM 24 (ukmo_hadgem1), GCM 18 (miub_echo_g), GCM 18 (miub_echo_g), GCM 18 (miub_echo_g), GCM 11 (giss_model_e_r), GCM 11 (giss_model_e_r), GCM 15 (ipsl_cm4), GCM 10 (giss_model_e_h), GCM 9 (giss_aom) are recommended respectively when simulating the inter-decadal temperature trends in the same regions. The results have shown that each AR4 GCM performs differently in different regions of China, particularly with respect to precipitation.

Further research is required regarding simulation of the climate characteristics of China. This includes further assessment of the accuracy of AR4 GCMs (by considering other climate variables and skill score metrics), application of the homogenization techniques, and using uncertainty analyses and multi-model ensemble predictions to improve the reliability of the model outputs.
